# Globus Pallidus Iron Relates to Cognitive Impairment in Alzheimer's Disease: Evidence From MRI‐Based Meta‐Analysis

**DOI:** 10.1111/nyas.70078

**Published:** 2025-12-04

**Authors:** Marthe Mieling, Clara Wiskow, Nico Bunzeck

**Affiliations:** ^1^ Department of Psychology University of Lübeck Lübeck Germany; ^2^ Center of Brain, Behavior and Metabolism University of Lübeck Lübeck Germany

**Keywords:** Alzheimer's disease, basal ganglia, cognitive performance, iron, MRI, meta‐analysis

## Abstract

Iron is essential for brain metabolism and cognitive functioning, but excessive levels during healthy and pathological aging can have detrimental effects. Although this notion was supported by several single studies, meta‐analytic evidence in Alzheimer's disease (AD) is still scarce. Therefore, we performed a meta‐analysis of 23 MRI experiments with, in total, 715 AD patients and 1130 healthy controls (HC). All studies employed iron sensitive markers in basal ganglia structures, thalamus, and hippocampus, together with the Mini‐Mental‐Status‐Examination (MMSE) to quantify cognitive performance. In all regions of interest, significantly higher iron levels were present in people with AD compared to HC, with the most pronounced effects in the putamen followed by the caudate. Importantly, only globus pallidus iron levels were negatively correlated with MMSE performance in AD patients. Our results provide unique evidence that increases in iron levels, especially within basal ganglia structures, which provide a hub for cognitive information processing, are a characteristic hallmark of AD.

## Graphical abstract

Iron is crucial for brain functioning, but excessive levels can harm brain health. While previous studies support this, meta‐analytic evidence in Alzheimer's disease (AD) is limited. We performed a meta‐analysis of 23 MRI studies with 715 AD patients and 1130 healthy controls. Higher iron levels in the basal ganglia were found in AD, with globus pallidus iron levels negatively correlating with cognitive performance, emphasizing iron's role in AD.

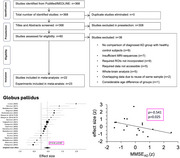

## 1 Introduction

Non‐heme iron is essential for several metabolic processes, ensuring normal brain functioning and behavior [1, 2]. However, increased cerebral iron levels are reported not only in healthy older adults [3, 4] but also in patients suffering from Alzheimer's disease (AD) and mild‐cognitive impaired (MCI) [e.g., 5, 6, 7, 8]. Therefore, regionally specific excessive iron could lead to cognitive decline [9, 10] and structural brain atrophy [10]. In fact, iron level increases have been linked with cognitive abilities, as measured with the Mini‐Mental‐Status‐Examination (MMSE) or Montreal‐Cognitive‐Assessment (MoCa) [10, 11, 12, 13]. Moreover, several post‐mortem and in vivo studies have provided evidence for a co‐localization of iron and tau [9, 14, 15] as well as amyloid pathology [16, 17] suggesting a direct relationship. While iron promotes oxidative stress [18, 19] and aggregation of amyloid‐beta peptide [20], they both (iron and oxidative stress) mediate amyloid‐beta toxicity [21]. Conversely, iron promotes tau phosphorylation [22] and the aggregation of hyperphosphorylated tau into neurofibrillary tangles [23, 24].

A previous post‐mortem meta‐analysis, published in 2014, revealed higher iron levels in the putamen, caudate nucleus, globus pallidus, amygdala, cingulate cortex, as well as the frontal, parietal, and temporal lobes in AD patients compared to healthy controls (HC) [25]. While this is an important finding, in vivo neuroimaging methods potentially allow for a much clearer link to cognitive decline as well as novel insights into the basis of paramagnetic brain iron [26, 27]. For instance, most studies comparing iron levels of AD versus HC using magnetic resonance imaging (MRI) reported significant iron level increases in basal ganglia structures, the amygdala, thalamus, and hippocampus [e.g, 6, 7, 28, 29, 30], which was confirmed by a recent neuroimaging meta‐analysis [31]. However, the latter focused only on original studies with quantitative susceptibility mapping (QSM) and did not focus on cognition; therefore, meta‐analytic evidence, especially concerning a possible relationship of increased iron levels and impaired cognitive abilities in AD, is scarce.

To this end, we performed random‐effects meta‐analyses on previously published data using iron‐sensitive MRI, such as T2*, T2′, R2*, R2′, field‐dependent *R*_2_ increase (FDRI), susceptibility‐weighted imaging (SWI), and QSM [26, 32, 33]. All included studies reported a comparison of AD patients versus age‐matched HC for either one or more brain regions: putamen, caudate nucleus, globus pallidus, hippocampus, and thalamus [e.g., 6, 7, 25, 28, 29, 30]. For every brain region, a single random‐effects model was calculated, and, in a subsequent step, the relationship between iron levels and cognitive performance (i.e., MMSE scores) in AD patients was investigated via linear regression.

## 2 Methods

### 2.1 Literature Search and Selection

**FIGURE 1:**
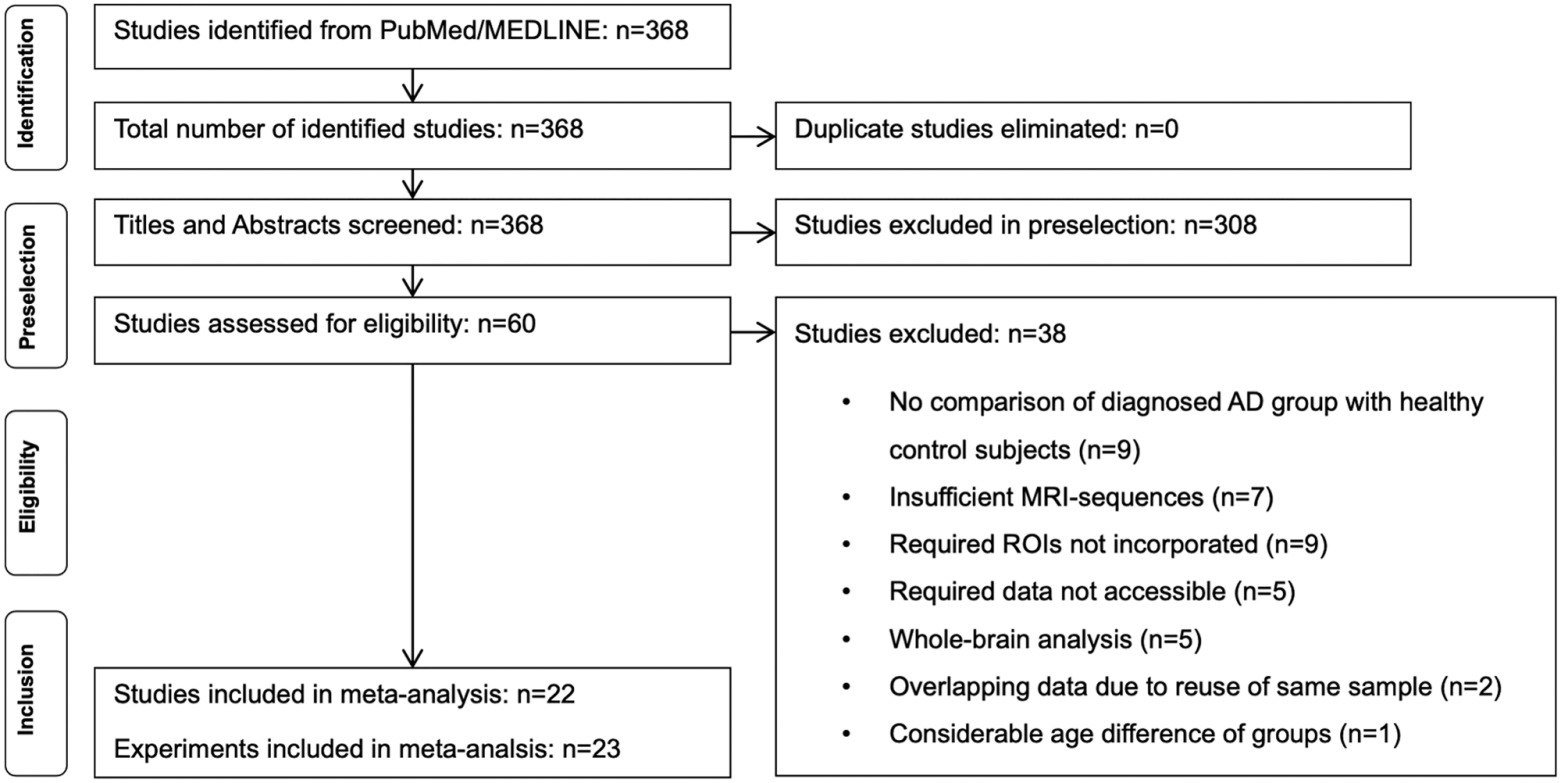
Flowchart of the steps performed in the meta‐analysis.

This study followed the PRISMA (Preferred Reporting Items for Systematic Reviews and Meta‐Analyses) 2020 guidelines [34] (see Figure 1). In detail, a systematic literature search was performed at https://pubmed.ncbi.nlm.nih.gov, which included PubMed and MEDLINE databases, by using the search terms “MRI” AND “iron” AND “Alzheimer” OR “MRI iron Alzheimer” to find original MRI studies on brain iron in AD patients and healthy age‐matched controls (see below for details on MRI sequences). The search included all potentially relevant and published studies until June 10, 2025—there were 368 original studies assessed in our preselection. Subsequently, titles and abstracts were screened, and as a result, 308 articles were excluded since they did not meet our inclusion criteria. The remaining 60 original studies were examined in more detail by two independent researchers (M.M. and C.W.) based on a full‐text reading with regard to the inclusion and exclusion criteria. An additional literature search was performed in June 2025 as part of a peer review process using Scopus (https://www.scopus.com), which revealed 360 original studies and did not yield any additional relevant articles.

The inclusion criteria comprised (1) AD patients diagnosed according to well‐established criteria; (2) the comparison of AD patients to HC; (3) no considerable age difference between AD patients and HC; (4) the data analysis was based on predetermined regions of interest (ROIs); (5) namely one or more of the following ROIs: putamen, caudate nucleus, globus pallidus, hippocampus, or thalamus; and (6) an iron‐sensitive MRI‐sequence was used, for instance, T2*, T2′, R2*, R2′, FDRI, SWI, and QSM. Articles were excluded based on the following criteria: (1) using T2 or R2 MRI sequence [26, 32, 35], (2) review articles, (3) post‐mortem studies, (4) animal models, (5) re‐analysis of previously used data (i.e., repeated use of the same sample), and (6) missing data information. If a study included multiple independent experiments contributing to a single analysis [36], these were incorporated and treated as  separate experiments. If necessary, a third researcher (N.B.) was consulted.

The final selection of included articles and study‐related information can be found in Table 1. Cross‐checking with the original literature prevented potential sample overlap between the studies. This refers to sample characteristics, author affiliations, and scanning locations.

**TABLE 1:** Overview of the experiment characteristics of all included experiments.

Authors and year	Method	Participants										Effect size (*d*)				
		AD						HC				PUT	CN	GP	HIP	THAL
		Group description	Diagnosis	N	Age (mean ± SD)	Sex (f/m)	MMSE	N	Age	Sex (f/m)	MMSE					
Ayton et al. (2017) [5]	QSM; 3T	Probable/possible AD	NINCDS‐ADRDA	19	—	—	—	64	—	—	—	—	0.501	—	−0.557	—
Bartzokis (2000) [92]	FDRI; 0.5T. 1.5T	Probable/possible AD	NINCDS‐ADRDA	31	75.6 ± 6.7	1.214	17.3 ± 7.39	68	68.8 ± 5.3	0.889	28.4 ± 0.92	0.677	0.652	0.182	—	—
Bartzokis (1994) [93]	FDRI; 0.5T. 1.5T	Probable AD; mild‐to‐moderate AD	NINCDS‐ADRDA	5	72.6 ± 3.97	0	17.8 ± 5.45	8	72.5 ± 4.5	0	28.6 ± 1.19	1.034	1.239	1.261	—	—
Bartzokis et al. (2004) [64]	FDRI; 0.5T. 1.5T	Probable/possible AD; onset >70 years	NINCDS‐ADRDA	10	81.6 ± 3.4	0	19.4 ± 5.4	36	68.7 ± 4.9	—	—	0.52	0.55	−0.296	—	—
Chen et al. (2024) [66]	QSM	Probable/possible AD	NINCDS‐ADRDA	30	68.5 ± 3.86	2.333	19.77 ± 4.97	26	65.5 ± 3.4	2.714	27.96 ± 1.64	—	—	—	−0.278	0.136
Chiang et al. (2022)[65]	QSM; 3T	Cognitively impaired	Exclusion of other etiologies; neurological examination. Neuropsychological assessment. Laboratory data. MRI	68	72 ± 8.4	1.519	—	32	69 ± 9.9	2.556	—	0.52	−0.07	−0.562	0.197	0.212
Cogswell et al. (2021) [39]	QSM; 3T	Amnestic dementia	NIA‐AA	56	68; IQR: 61–77	1.074	—	296	69 IQR: 59–76	0.762	—	0.46	−0.154	0.438	—	−0.828
Damulina et al. (2020) [28]	R2*; 3T	Probable/possible AD	DSM‐IV. NINCDS‐ADRDA	100	73 ± 9	1.381	22; IQR: 18.5–25	100	73 ± 9	1.5	28; IQR: 27–29	0.378	0.495	0.243	0.04	−0.054
Ding et al. (2009) [29]	Phase; 1.5T	Probable AD	NINCDS‐ADRDA	26	70.96 ± 8.55	2.25	16.0 ± 3.9	24	69.40 ± 11.38	1.667	29.4 ± 1.0	0.691	—	0.93	0.645	—
Du et al. (2018) [7]	QSM; 3T	Mild‐to‐moderate AD	NINCDS‐ADRDA	30	68.3 ± 6.6	2.333	20.4 ± 2.4	30	66.2 ± 7.8	2	28.0 ± 1.3	0.571	0.574	−0.013	—	−0.319
Gao et al. (2017) [50]	SWI; 3T	Probable AD	DSM‐IV. NINCDS‐ADRDA	30	74.83 ± 4.52	1.5	17.76 ± 4.15	30	72.86 ± 5.75	1.308	28.73 ± 1.11	1.849	0.63	0.555	0.628	—
Guan et al. (2022) [43]	QSM; 3T	Probable AD	NINCDS‐ADRDA	51	68.7 ± 8.7	0.889	19.6 ± 3.9	189	61.4 ± 7.8	1.423	—	1.459	0.901	0.311	—	—
Huang et al. (2023) [41]	QSM; 3T	AD	NIA‐AA	43	62.63 ± 8.10	2.301	20.3 ± 1.89	27	58.70 ± 8.99	1.7	27.30 ± 2.20	0.591	0.793	0.4	—	—
Kim et al. (2017) [6]	QSM; 3T	Probable AD; mild AD	NINCDS‐ADRDA	19	69.79 ± 10.27	8.5	17.37 ± 3.42	19	65.37 ± 6.26	5.333	28.16 ± 1.89	2.765	—	1.283	5.799	4.761
Kuchcinski (2022) [30]	QSM; 3T	EOAD; probable AD; typical AD (tADMRI)	NIA‐AA	34	60.6 ± 4	1.267	15.8 ± 6	43	62.0 ± 4.3	1.867	—	0.876	0.274	0.915	0.211	1.9
Li et al. (2020) [11]	QSM; 3T	Probable AD	NIA‐AA	22	71.5 ± 8.4	1.444	18.9 ± 3.4	25	69.3 ± 5.2	1.5	29.7 ± 0.6	2.376	1.433	2.47	0.127	0.739
Liu et al. (2021) [12]	QSM; 3T	AD	NINCDS‐ADRDA	30	68.37 ± 6.734	2.75	19.8 ± 3.925	19	66.68 ± 8.564	2.8	28 ± 1.856	0.516	2.064	0.139	—	—
Moon et al. (2012) [49]	T2*; 3T	AD	NINCDS‐ADRDA	21	72.1 ± 6.5	2.5	21.2 ± 3.8	21	68.9 ± 5.3	4.25	28.0 ± 1.2	0.76	—	−0.462	—	0.9
Qin et al. (2011) [45]	R2'; 1.5T	Probable AD	NINCDS‐ADRDA	15	69.8	1.143	17.3	15	70.0	1.143	30	0.516	1.077	—	1.235	0.177
Tiepolt et al. (2020) [40]	QSM; 3T	Cognitive impairment + Aβ PET‐positive	NINCDS‐ADRDA	16	69 ± 9	3	25 ± 2	11	65 ± 3	1.75	30 ± 1	1.246	0.144	−0.07	0.487	—
Wang et al. (2014) [13]	SWI; 3T	Probable AD; age 60–70 years	NINCDS‐ADRDA	20	—	0.818	20.3 ± 2.98	19	—	0.9	28.22 ± 0.97	0.134	−0.303	0.702	—	—
Wang et al. (2014) [13]	SWI; 3T	Probable AD; age 70–80 years	NINCDS‐ADRDA	19	—	0.818	20.3 ± 1.89	10	—	0.9	27.84 ± 2.47	0.467	0.262	0.846	—	—
Wang et al. (2013) [51]	SWI; 3T	Probable AD	NINCDS‐ADRDA	20	73.37 ± 9.81	0.758	21.15 ± 1.23	18	70.52 ± 6.91	1.087	28.22 ± 0.87	—	0.66	—	1.173	0.951

Abbreviations: AD: Alzheimer's disease; CN: caudaute nucleus; GP: globus pallidus; HC: healthy controls; HIP: hippocampus; MMSE: Mini‐Mental‐State‐Examination; PUT: putamen; THAL: thalamus.

### 2.2 Participants

Participants were included and grouped based on their diagnoses explicitly reported in the original articles. The AD group met the diagnostic criteria for probable or possible AD, mostly in alignment with the National Institute of Neurological and Communicative Disorders and Stroke‐Alzheimer's Disease and Related Disorders Association (NINCDS‐ADRDA) or the National Institute on Aging and Alzheimer's Association (NIA‐AA) [37]. Since additional information could enhance diagnostic accuracy [38], we assessed how many studies reported data on amyloid, tau, or *APOE4*. However, the majority did not provide this information (amyloid: *n* = 5 [6, 9, 30, 39, 40], tau: *n* = 2 [30, 39], *APOE4*: *n* = 4 [9, 30, 39, 41]), so we were unable to incorporate these biomarkers into our analysis.

Moreover, in all original articles, AD patients and HC were matched with regard to sex and age, which we further tested across studies using independent *t*‐tests. Participants were scanned at various scanner field strengths. For instance, FDRI was applied at field strengths of 0.5 Tesla and 1.5 Tesla, and phase imaging and R2′ were performed using a 1.5 Tesla scanner, while all other sequences were implemented at a field strength of 3 Tesla (see Table 1).

### 2.3 Meta‐Analyses

#### 2.3.1 Overview

Our analyses were based on the following steps that will be explained in more detail below. First, the effect sizes (Cohen's *d*) of group differences in iron levels were calculated for all available ROIs on the basis of their mean values and standard deviations (SD). Second, five random‐effects meta‐analyses were computed separately for each ROI (putamen, caudate nucleus, globus pallidus, hippocampus, and thalamus). Third, we assessed a possible relationship between iron level differences between groups (effect sizes) and cognitive performance (MMSE). Our calculations are available at the Open Science Framework (OSF, https://tinyurl.com/3u9n6cwh).

#### 2.3.2 Effect Size Calculation—Cohen's *d*

The effect size (Cohen's *d*), bias‐corrected for small sample sizes, was calculated for each ROI and original study based on mean values and SD [42]. In three studies [28, 39, 43], the median and interquartile range (IQR) were reported instead of the mean and the standard deviation. Therefore, the median was converted to a mean, and the IQR was converted to SD [44]. In five studies [7, 12, 39, 43, 45], the mean and SD of the MRI data, or median and IQR, respectively, were not available in numerical data, but they were shown in figures. Therefore, PlotDigitizer [46] was used to extract the data [47]. For studies reporting separate values for the left and right hemispheres (*n* = 12), we calculated the mean of the two values to obtain a single average score. The corresponding SDs were pooled using the standard formula for combining two independent variances, ensuring consistent data aggregation across studies [48].

MRI‐based markers of iron levels need to be carefully interpreted [26]. The interpretation of signal values in relation to iron load was confirmed for each included study by reviewing the original publication. For some markers, high values indicate high iron levels (e.g., R2* [28], QSM [40], see Table 1 for all MRI methods used in the original studies), but for others, low values indicate high iron levels (here T2* in milliseconds [49], phase values measured as radians [29], SWI [13, 50, 51]). Therefore, effect sizes had to be negated (multiplied by −1) when they were based on phase image [29], SWI [13, 50, 51], and T2* [49]. As a result, positive effect sizes indicated higher iron levels in AD compared to HC, while negative effect sizes indicate higher iron levels in HC compared to AD.

Finally, individual effect sizes were used to calculate the mean overall effect across all studies. Values of 0.2, 0.5, and 0.8 indicated small, medium, and large effects, respectively [48].

#### 2.3.3 Random‐Effects Models

Following Borenstein et al. [52], for each ROI, a single random‐effects model was calculated. To ensure high overall effect precision, individual effect sizes were weighted by each experiment's inverted total variance. Significance testing of the weighted overall mean effect size (d*) was based on *z*‐scores and corresponding *p* values (two‐tailed). We also calculated 95% confidence intervals and *I* ^2^ statistics to quantify heterogeneity [52, 53]. *I* ^2^ indicates inconsistency across experiments and represents the percentage of heterogeneity within the total variance of the primary studies [52, 53]. It can be compared across different meta‐analyses and ranges from 0 to 100. More specifically, it differentiates between low (25%), moderate (50%), and high (75%) heterogeneity [53]. Moreover, funnel plots were created, and Egger's regression tests were performed [54] to address possible publication biases [55, 56]. A funnel plot represents each experiment's effect sizes against standard errors, with an asymmetry indicating a publication bias [56, 57, 58]. Egger's regression test detects publication bias based on the funnel plot by assessing the relationship between standardized effect estimates and their standard error [54, 56]. Here, we generated funnel plots and conducted Egger's regression test using effect sizes and results from the random effect models within “Meta‐Essentials: Workbooks for meta‐analysis” (Version 1.4) [59].

Outliers (effect size) were identified as data points exceeding 1.5 times the IQR above the third or below the first quartile [60]. To enhance the robustness of the findings, the meta‐analyses were rerun after identifying and excluding any outliers detected.

##### 2.3.3.1 Relationship Between Brain Iron Deposition and Cognition in Alzheimer's Disease

To investigate possible links between iron accumulations in AD and cognitive impairment, five correlation analyses were conducted for each region‐specific meta‐analysis separately in Jamovi (2.3.21) [61]. Here, effect sizes for our five ROIs, indicating iron level increases in AD compared to HC, were correlated with the MMSE mean scores from the AD groups reported in the original studies. Specifically, we used *z*‐scored MMSE values from the AD group and iron effect sizes (i.e., AD vs. HC) in combination with Spearman's rank correlation (Spearman's Rho) due to the rather small sample size and since it is more robust against outliers [62]. In an additional analysis, we also computed MMSE effect sizes (for AD vs. HC) and correlated them with iron effect sizes (i.e., AD vs. HC). Since we predicted a negative correlation between iron levels and cognitive performance and different studies were included in the analyses, we did not apply a correction for multiple comparisons in this analysis. We also compared the correlation coefficients between regions using independent sample comparisons implemented in  *cocor* [63].

## 3 Results

### 3.1 Study Characteristics

Overall, we included 715 AD patients and 1130 HC from 22 studies with 23 experiments (see Figure 1). One study investigated two independent age groups of AD patients and HC, representing two individual analyses [36]. Therefore, this study was included in the meta‐analyses with both experiments [13]. For the separate five ROI meta‐analyses, 20 experiments were included for the putamen, 19 for the caudate nucleus, 19 for the globus pallidus, 12 for the hippocampus, and 11 for the thalamus (see Table 1 for an overview of the included experiments with more detailed information).

Although individual studies reported age matched AD and HC groups, a *t*‐test across studies revealed significant age differences (*t*(38) = −2.22, *p* = 0.032). Accordingly, AD patients were slightly older (mean = 70.56 years, SD = 4.47) than HC (mean = 67.64 years, SD = 3.82). Note that three experiments from two studies [5, 13] did not report detailed information on age and could, therefore, not be included in our analysis. Mean sex ratios (female/male) of AD patients and HC were 1.81 (SD = 1.71) and 1.81 (SD = 1.21), respectively, with no significant differences across studies as assessed by a Mann–Whitney *U* test due to the violation of normal distribution (*U* = 210, *p* = 0.618). One study [9] did not report the sex for both groups and another [64] solely for the AD group. Both studies were not included in our analysis.

### 3.2 Random Effects Meta‐Analyses

#### 3.2.1 Putamen

**FIGURE 2:**
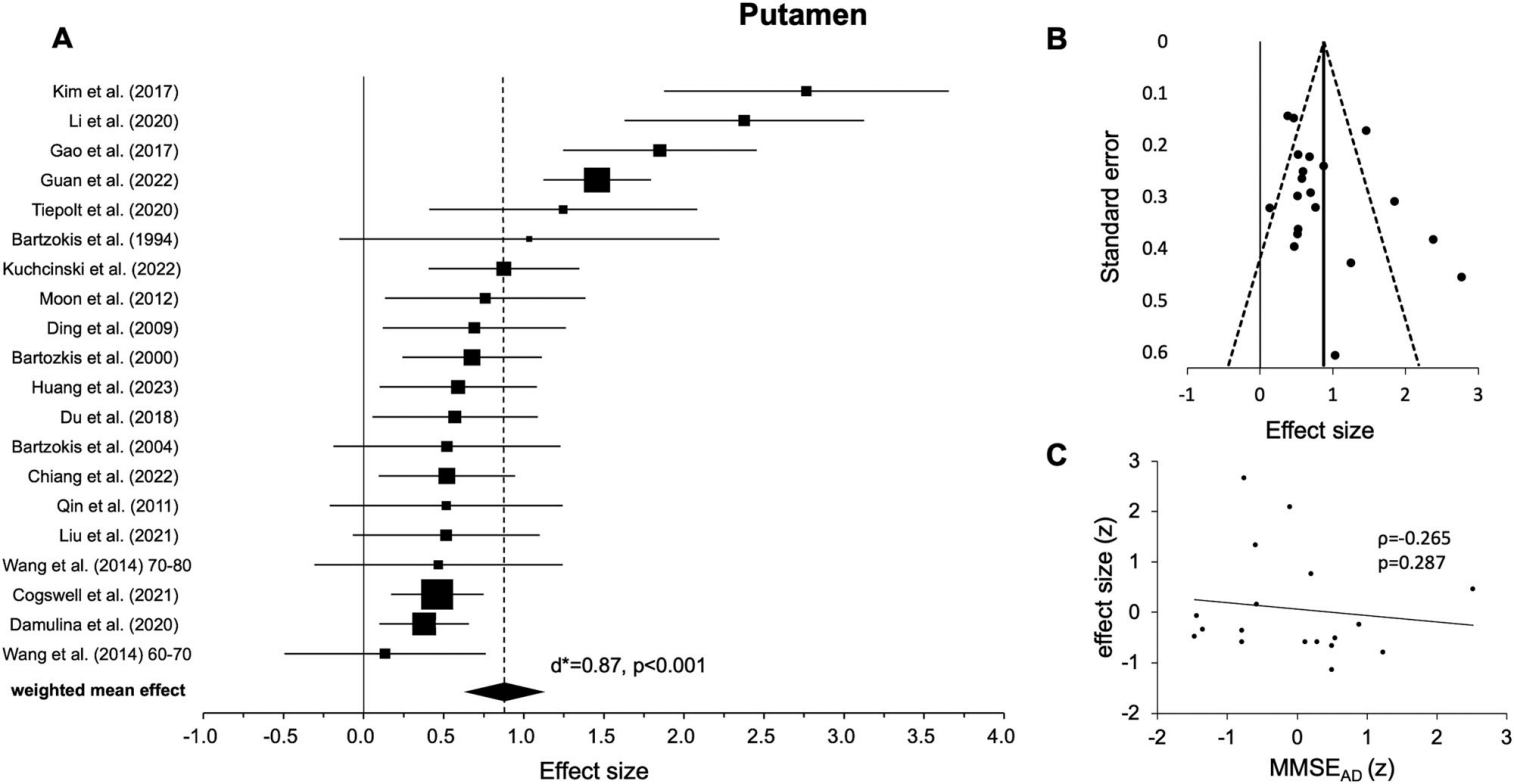
Results for the putamen. (A) Forest plot of group comparisons for Alzheimer's disease (AD) patients versus healthy controls (HC). Squares represent the computed effect sizes of the original studies, the square's size indicates the relative size of the sample studied, and the diamond represents the weighted mean effect. The width of the diamond as well as the horizontal lines of the squares indicate an experiment's 95% confidence interval. (B) Funnel plot: the dots mark the individual experiment (*k* = 20), the dashed line the 95% confidence interval, and the vertical straight line the overall effect. (C) Spearman correlation of iron differences between both groups (AD vs. HC, effect size) and mean Mini‐Mental‐State‐Examination (MMSE) scores (AD, *k* = 18).

The random‐effects model for the putamen included 20 experiments from 19 studies (see Table 1). In all original experiments, the weighted mean effects were positive and statistical testing across studies revealed a highly significant positive effect indicating higher iron levels in AD versus HC (d* = 0.87, *p* < 0.001, Figure 2A, Table 2). Further testing revealed high heterogeneity of *I* ^2^ = 78.39%. After removing two outliers [6, 11], the weighted mean effect was still highly significant, but it showed a slightly reduced effect size (d* = 0.72, *p* < 0.001, *I* ^2^ = 65.47).

**TABLE 2:** Overview of the results of the region of interests (ROI) random‐effects models for comparing Alzheimer's disease patients versus healthy controls.

*Meta‐analytic information*							
Brain regions	*k*	d*	CI (95%) *_d_*	*Z*	*p*	*I* ^2^	CI (95%) *_I_* ^2^
Putamen	20	0.87	0.62–1.13	6.76	<0.001	78.39	67.17 – 85.78
Caudate nucleus	19	0.58	0.34–0.81	4.74	<0.001	75.84	62.39 – 84.48
Globus pallidus	19	0.44	0.19–0.69	3.41	0.001	78.61	67.17–86.06
Hippocampus	12	0.60	0.15–1.04	2.65	0.008	87.65	80.28–92.26
Thalamus	11	0.67	0.09–1.26	2.27	0.023	93.61	90.43–95.74

*Note*: Displayed are the number of experiments (*k*) included, the weighted mean effects (d*) of each ROI, 95% confidence intervals (CI), *Z*‐values, and *p* values as well as the heterogeneity indicators *I* ^2^ and their 95% CIs.

#### 3.2.2 Caudate Nucleus

**FIGURE 3:**
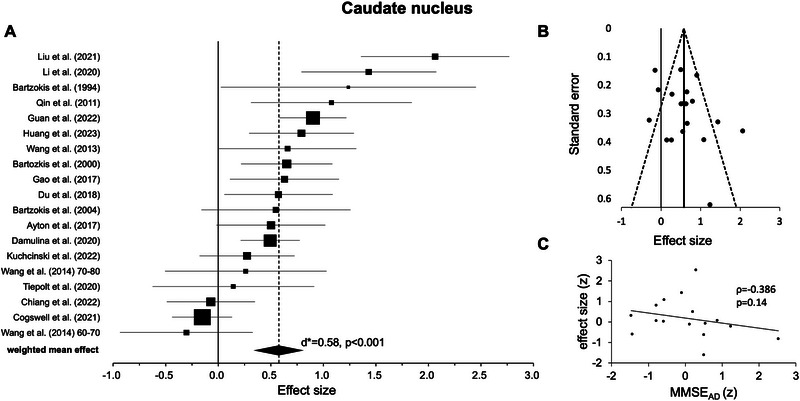
Results for the caudate nucleus. (A) Forest plot of group comparisons for Alzheimer's disease (AD) patients versus healthy controls (HC). Squares represent the computed effect sizes of the original studies, the square's size indicates the relative size of the sample studied, and the diamond represents the weighted mean effect. The width of the diamond and the horizontal lines of the squares indicate an experiment's 95% confidence interval. (B) Funnel plot: the dots mark the individual experiment (*k* = 19), the dashed line the 95% confidence interval, and the vertical straight line the overall effect. (C) Spearman correlation of iron differences between groups (AD vs. HC, effect size) and mean Mini‐Mental‐State‐Examination (MMSE) scores (AD, *k* = 16).

The random‐effects model for the caudate nucleus included 19 independent experiments from 18 original studies (see Table 1). While most weighted mean effects were positive, three experiments [13, 39, 65] showed negative effects. However, statistical testing across experiments revealed a highly significant positive effect, again suggesting higher iron levels in AD versus HC (d* = 0.58, *p* < 0.001, Figure 3A, Table 2). Further testing revealed high heterogeneity of *I* ^2^ = 75.84%. After removing one outlier [12], the effect remained highly significant (d* = 0.50, *p* < 0.001, *I* ^2^ = 68.93%).

#### 3.2.3 Globus Pallidus

**FIGURE 4:**
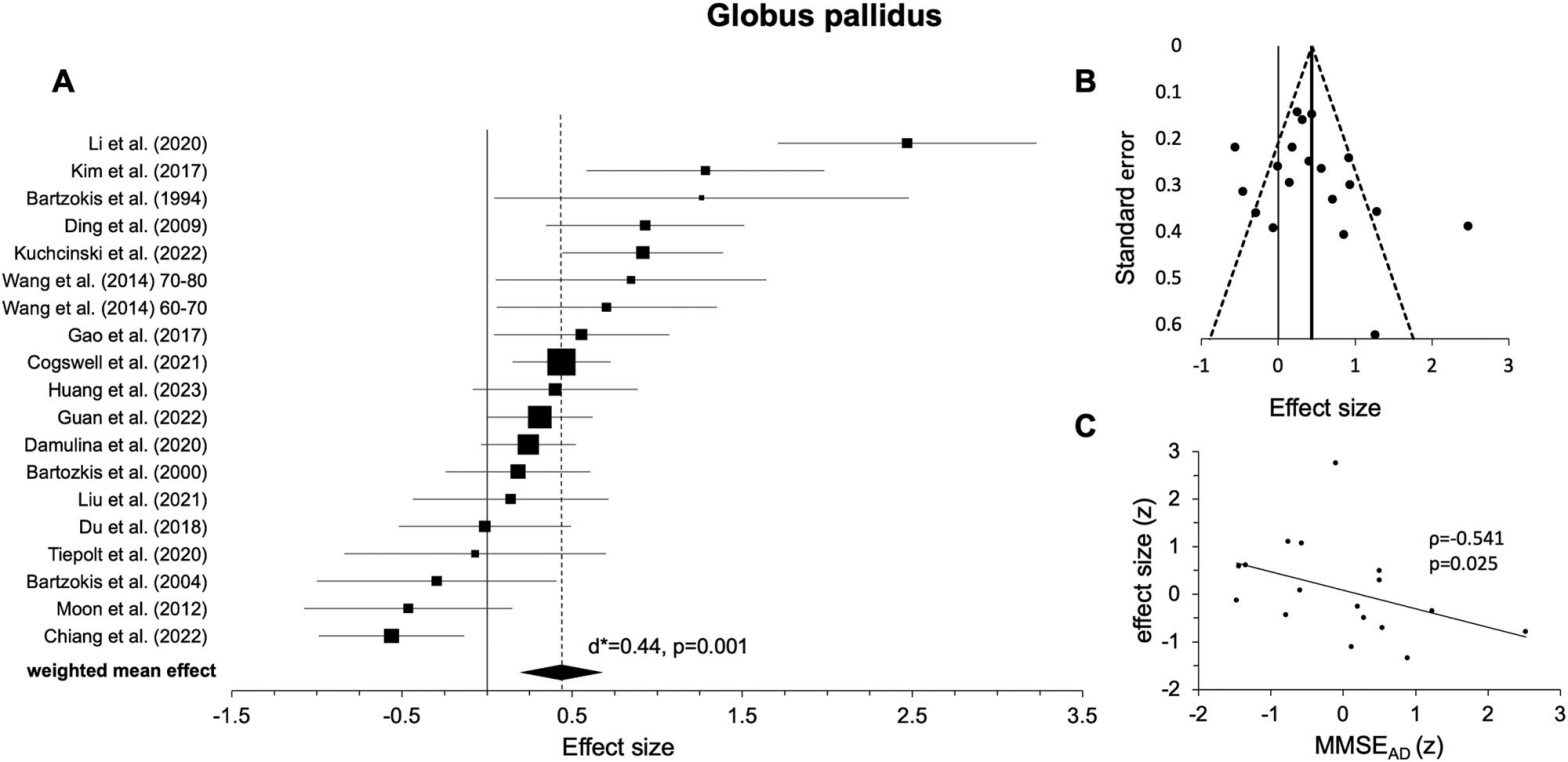
Results for the globus pallidus. (A) forest plot of group comparisons for Alzheimer's disease (AD) patients versus healthy controls (HC). Squares represent the computed effect sizes of the original studies, the square's size indicates the relative size of the sample studied, and the diamond represents the weighted mean effect. The width of the diamond and the horizontal lines of the squares indicate an experiment's 95% confidence intervals. (B) Funnel plot: the dots mark the individual experiment (*k* = 19), the dashed line the 95% confidence interval, and the vertical straight line the overall effect. (C) Spearman correlation of iron differences between groups (AD vs. HC, effect size) and mean Mini‐Mental‐State‐Examination (MMSE) scores (AD, *k* = 17).

For the globus pallidus, 19 independent experiments from 18 studies were included in the random‐effects model (see Table 1). Except from five experiments [7, 40, 49, 64, 65], all others showed positive weighted mean effects. Statistical analysis revealed a highly significant effect indicating higher iron levels in AD versus HC (d* = 0.44, *p* = 0.001, Figure 4A, Table 2); again, heterogeneity was rather high (*I* ^2^ = 78.61%). After excluding one outlier [11], the results remained statistically significant (d* = 0.33, *p* = 0.002, *I* ^2^ = 68.34%).

#### 3.2.4 Hippocampus

**FIGURE 5:**
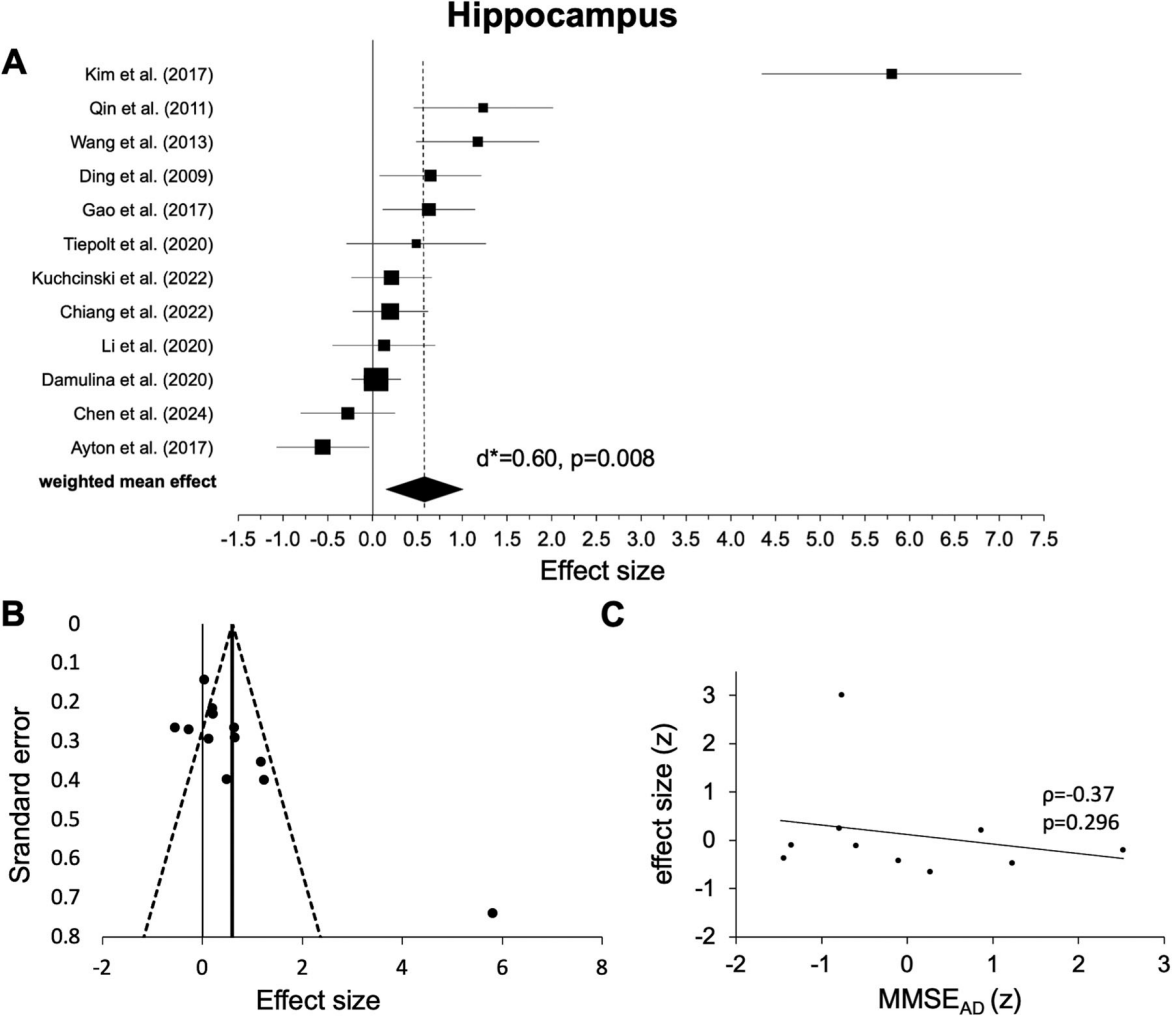
Results for the hippocampus. (A) Forest plot of group comparisons for Alzheimer's disease (AD) patients versus healthy controls (HC). Squares represent the computed effect sizes of the original studies, the square's size indicates the relative size of the sample studied, and the diamond represents the weighted mean effect. The width of the diamond and the horizontal lines of the squares indicate an experiment's 95% confidence intervals. (B) Funnel plot: the dots mark the individual experiment (*k* = 12), the dashed line the 95% confidence interval, and the vertical straight line the overall effect. (C) Spearman correlation of iron differences between groups (AD vs. HC, effect size) and mean Mini‐Mental‐State‐Examination (MMSE) scores (AD, *k* = 10).

The random‐effect model for the hippocampus included only 12 experiments from 12 studies (see Table 1). All but two experiments [9, 66] showed positive mean weighted effects and, across experiments, statistical testing revealed a highly significant positive effect. Again, this indicates higher iron levels for AD versus HC (d* = 0.60, *p* = 0.008, Figure 5A, Table 2); heterogeneity was high (*I* ^2^ = 87.65%). After excluding one outlier [6], the results remained significant, but the effect size was rather small (d* = 0.30, *p* = 0.036, *I* ^2^ = 69.29%).

#### 3.2.5 Thalamus

**FIGURE 6:**
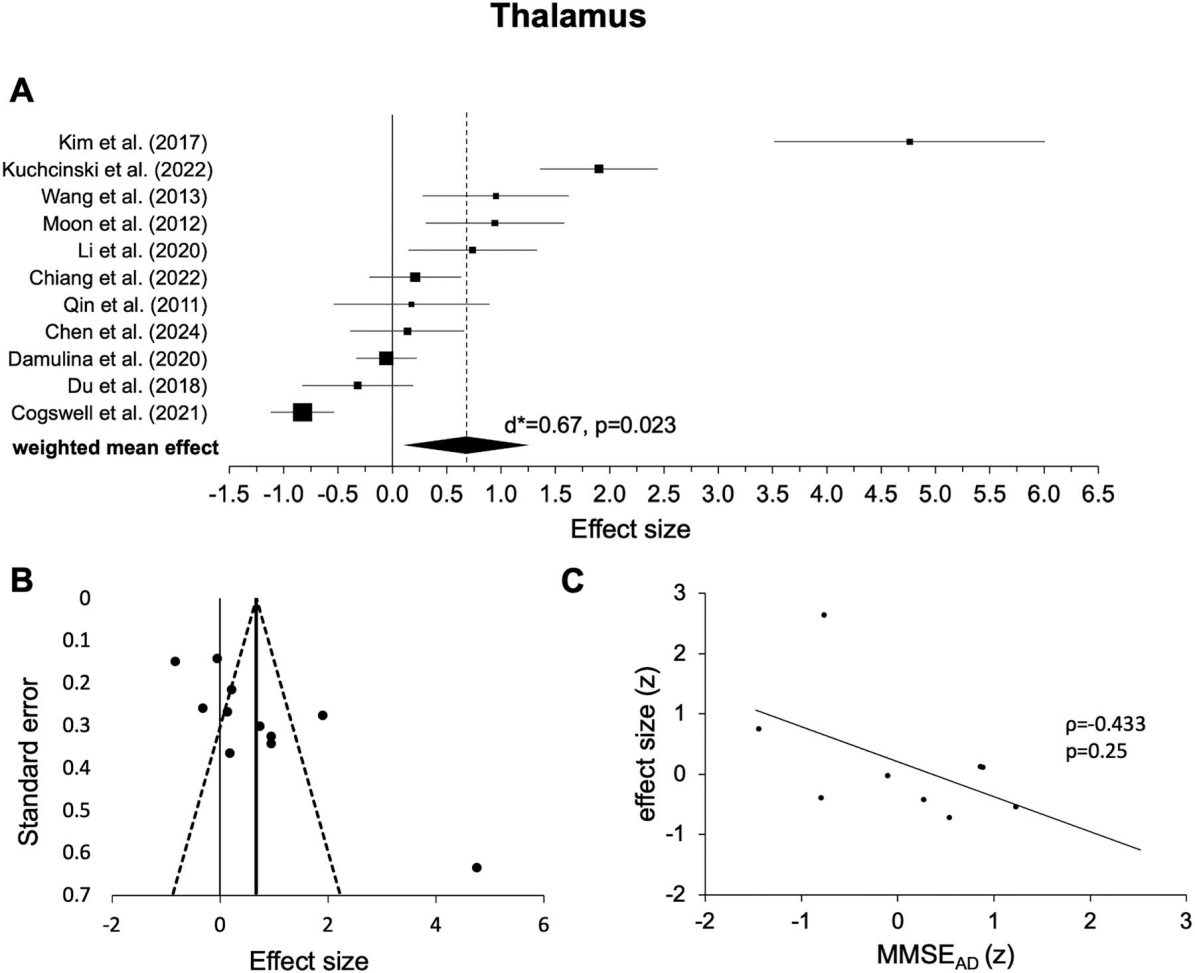
Results for the thalamus. (A) Forest plot of group comparisons for Alzheimer's disease (AD) patients versus healthy controls (HC). Squares represent the computed effect sizes of the original studies, the square's size indicates the relative size of the sample studied, and the diamond represents the weighted mean effect. The width of the diamond and the horizontal lines of the squares indicate an experiment's 95% confidence intervals. (B) Funnel plot: the dots mark the individual experiment (*k* = 11), the dashed line the 95% confidence interval, and the vertical straight line the overall effect. (C) Spearman correlation of iron differences between groups (AD vs. HC, effect size) and mean Mini‐Mental‐State‐Examination (MMSE) scores (AD, *k* = 9).

The random‐effects model for the thalamus involved only 11 experiments from 11 studies (see Table 1). While three weighted means were negative [7, 28, 39], the remaining eight were positive. Across experiments, statistical testing revealed a significant positive effect (d* = 0.67, *p* = 0.023, Figure 6A, Table 2), also indicating higher iron levels in AD versus HC. Again, heterogeneity was high (*I* ^2^ = 93.61). After excluding one outlier [6], the analysis revealed no significant effect (d* = 0.36, *p* = 0.15), and still a high heterogeneity of the data (*I* ^2^ = 91.23%).

#### 3.2.6 Publication Bias

Upon visual inspection, data points in most ROIs showed a symmetric distribution around the weighted mean effect (Figures 2–6B), indicating no publication bias. However, for the hippocampus (Figure 5B) and thalamus (Figure 6B), the funnel plots showed some degree of asymmetry. In line with this impression, Egger's regression test revealed a significant effect for the hippocampus (*p* < 0.001) and thalamus (*p* < 0.001), indicating a publication bias, but not for the putamen (*p* = 0.110, Figure 2B), caudate nucleus (*p* = 0.157, Figure 3B), and pallidum (*p* = 0.112, Figure 4B).

#### 3.2.7 Correlations Between Brain Effect Sizes and MMSE in Alzheimer's Disease

Correlation analyses of the iron effects sizes (AD vs. HC, z‐scored) and MMSE mean group performance (*z*‐scored) in AD revealed a significant negative effect only in the globus pallidus (*r*(15) = −0.541, *p* = 0.025, Figure 4C, Table 3). One data point was visually prominent (effect size [*z*] = 2.77) and met our exclusion criteria of exceeding 1.5 times the IQR above the third or below the first quartile. When excluding this case, the correlation remained significant (*p* = 0.026). For the other ROIs, the effects were not statistically significant (*p* > 0.05, Figures 2, 3, 5, and 6C; Table 3). Comparing the correlation coefficients from different regions did not reveal significant differences (*p* > 0.05).

**TABLE 3:** Correlations between the region‐of‐interests′ original studies′ effect sizes and the Mini‐Mental‐State‐Examination mean of the Alzheimer's disease group.

Brain regions	MMSE mean AD group		
	Spearman's Rho	*p* value	*k*
Putamen	−0.265	0.287	18
Caudate nucleus	−0.386	0.14	16
Globus pallidus	−0.541	0.025*	17
Hippocampus	−0.370	0.296	10
Thalamus	−0.433	0.250	9

*Note*: The correlations (Spearman's Rho) between the original studies' effect sizes for the brain regions (putamen, caudate nucleus, globus pallidus, hippocampus, and thalamus) with the Mini‐Mental‐State‐Examination (MMSE) mean of the Alzheimer's disease (AD) groups from the original studies. Presented are the *p* values (*<0.05), and *k* reflecting the number of included experiments (in that both MMSE scores for AD and the effect sizes were available). Data were *z*‐standardized before entering the analysis.

To investigate this effect further, we repeated the correlation analyses with iron effect sizes (AD vs. HC, z‐scored) and MMSE effect sizes (AD vs. HC, z‐scored). It also revealed a statistically significant effect (*r*(12) = −0.569, *p* = 0.037). Note, however, in this latter analysis, three experiments had to be excluded [30, 43, 64] due to missing MMSE information in the HC group. Comparing the correlational coefficients from different regions did not reveal significant effects (*p* > 0.05).

## 4 Discussion

We leveraged the power of a meta‐analytic approach to investigate the relationship between regional brain iron levels and cognitive abilities in AD. We employed data from 23 experiments encompassing 1845 participants, including 715 patients diagnosed with Alzheimer's disease and 1130 age‐matched HC. As expected, in AD iron levels were significantly higher in the putamen, caudate nucleus, and globus pallidus, but also the hippocampus and to a lesser extent in the thalamus. Importantly, iron accumulation in the globus pallidus, a basal ganglia hub with a critical role in several cognitive processes, was negatively associated with cognitive performance as measured using the MMSE. Therefore, our results provide unique evidence across a wide range of in vivo studies suggesting that iron accumulation in the basal ganglia is a characteristic feature in AD that contributes to cognitive decline.

Iron plays a central role in maintaining normal brain functioning, contributing to myelination [67, 68], neurotransmitter synthesis [69], and oxygen regulation [2]. However, excessive iron levels can induce oxidative stress and inflammation [69], demyelination [3], and ferroptosis, an iron‐dependent form of cell death [70], and therefore, promote the progression of AD. From a developmental perspective, brain iron accumulates over the lifespan [1, 4, 11, 71, 72, 73, 74] with inter regional variations and particularly high levels in the basal ganglia [1, 26, 75]. This pattern appears to reflect region specific needs in iron to meet metabolic demands [76]. The excessive iron deposition in AD may, therefore, closely relate to dysregulations in iron metabolism and transport [77], but also vascular hemorrhages and microbleeds [77, 78], as well as neuroinflammation [78]. Moreover, post‐mortem and in vivo studies revealed a co‐localization of iron and tau [9, 14, 15] as well as amyloid pathology [16, 17]. In line with this observation, a positive correlation of MRI susceptibility has been shown for both amyloid [16, 39] and tau [15, 39, 79] PET in the basal ganglia, underscoring the importance of iron increases in AD. These findings suggest that iron accumulation may contribute to neurodegeneration both directly and indirectly, by amplifying amyloid and tau pathology and by triggering iron‐dependent cell death pathways such as ferroptosis [80]. Clarifying the temporal and mechanistic interplay between these processes is a key objective for future research.

Our results of iron accumulations in the putamen, caudate nucleus, and globus pallidus are consistent with this prior research and a meta‐analysis investigating post‐mortem iron accumulations in these brain regions [25]. In comparison, the most pronounced effect was observed in the putamen, as suggested by a large weighted effect size [48], indicating a contribution by all original studies (Figure 2A and Table 1). Although still highly significant, the random‐effects model for the caudate nucleus showed a medium‐weighted effect size with all but three studies [13, 39, 65] contributing to the effect (Figure 3A and Table 1). Finally, the random‐effects model for the globus pallidus, again highly significant, had a rather small weighted effect size, and all but five studies [7, 40, 49, 64, 65] contributed to the effect (Figure 4A and Table 1). Taken together, for the investigated basal ganglia structure, putamen, caudate nucleus, and globus pallidus, almost all included studies reported higher iron levels in AD compared to HC, which led to highly significant effects in our meta‐analysis. Egger's regression and visual inspection of the funnel plots (Figures 2–4B) did not indicate any publication bias for these regions, which further underlines the robustness of our findings.

Outside the basal ganglia, we also observed significant effects in the hippocampus (Figure 5A) and the thalamus (Figure 6A), indicating higher iron levels in AD compared to HC. However, the thalamus effect did not remain significant after removing one outlier, and in the thalamus as well as the hippocampus, a publication bias was detected (Figures 5 and 6B). Together with small‐to‐moderate‐weighted effect sizes, this casts doubt on the reliability of the thalamus and hippocampus effect. However, both analyses were based on a small number of experiments (11 for the thalamus and 12 for the hippocampus), indicating a possible power problem. Yet, our finding is in line with a previous post‐mortem meta‐analysis that also did not reveal statistically significant iron effects in AD in the thalamus (*p* = 0.16) or hippocampus (*p* = 0.056) [25] (albeit with even fewer original experiments compared to our work, *k* > 8) [25]. Similarly, a recently published neuroimaging QSM meta‐analysis [31] found no significant iron effect in the thalamus (*p* = 0.17) and was also based on a smaller number of original experiments (*k* = 9) compared to our study.

Based on several single studies, we predicted a negative relationship between iron levels and cognitive performance in AD [10, 11, 12, 13]. This has been confirmed in the globus pallidus (Figure 4c), further indicating that local increases in iron levels lead to cognitive impairments not only in healthy older adults [3, 4] but also AD. From a mechanistic point of view, the pallidum has long been associated with motor functions, but it also plays a role in cognitive information processing [81]. In fact, the ventral pallidum is part of a polysynaptic pathway by which hippocampal novelty signals are relayed to dopaminergic neurons in the substantia nigra/ventral tegmental area (SN/VTA), which in turn back project to the hippocampus to support long‐term memory encoding [82, 83]. While iron is required for dopamine (DA) synthesis [69], an accumulation of it can impair DA production [69, 84]. Therefore, excessive iron levels within the bilateral pallidum may account for an imbalance of the hippocampus–SN/VTA loop, leading to impairments in declarative learning and memory, which is a central (but not the only) aspect in the MMSE. Further, basal ganglia structures and DA have been associated with motivation, decision‐making, and non‐declarative memory functioning [85], which may also contribute to MMSE performance. Indeed, a negative relationship between iron deposition in the globus pallidus and MMSE scores in 60‐ to 80‐year‐old AD patients has been reported before [13]. Taken together, our findings can be explained on the basis of previous work, and they further suggest that iron accumulation in the pallidum contributes to cognitive decline in AD.

A recent clinical trial using deferiprone for iron chelation found that, despite reduced hippocampal iron levels as measured by QSM, cognitive performance declined in amyloid‐positive patients with MCI and AD [86]. While this may initially appear inconsistent with our rationale, it points to a more complex and multifactorial role of brain iron in AD. In fact, elevated iron levels in AD may reflect an adaptive response, or alternatively, it could become sequestered within pathological aggregates, leading to regional accumulation alongside a state of functional iron deficiency [86]. Moreover, there may exist a harmful subset of iron contributing to disease progression that was not effectively targeted by deferiprone at the administered dose. Taken together, these observations support a role of iron in cognitive functioning in AD, and they further highlight the need for therapeutic strategies that carefully distinguish between toxic and functionally relevant iron.

Finally, some other limitations, which may guide future research, need to be considered. First, a formal comparison between correlations across regions did not reveal any significant differences. Thus, while globus pallidus iron levels in AD were associated with cognitive functioning (i.e., MMSE), the evidence is not conclusive regarding regional specificity. Second, iron measures were averaged from both hemispheres, streamlining our analysis but neglecting possible lateralization effects [87]. Third, we observed rather high heterogeneity in all random effect models [53], which, despite strict inclusion and exclusion criteria (see Section 2), could be driven by differences in MRI sequences and field strengths [26, 33, 88], regions of interest definitions, and sample characteristics (e.g., different stages of AD). To further substantiate our findings, however, all analyses were repeated without outliers, when necessary, and we also report empirical measures for potential publication biases (Figures 2–6B). Fourth, although all individual studies included age‐matched HC, across studies a slight but significant age difference was detected (Table 1), which might have biased the results. Fifth, all studies included used a cross‐sectional design, which allows for between‐group comparisons but, strictly speaking, not the description of developmental and disease‐specific changes. Longitudinal designs may help to address this important point. Sixth, in the original studies, participants were included solely based on their clinical status. Future work could be based on other approaches, especially the ATN framework, which includes markers of amyloid‐β (A), tau (T), and neurodegeneration (N) [38] or the *APOE4* genotype which also appears to be relevant for iron accumulation [80]. This would allow for the exploration of differences (or changes) in iron levels more precisely as well as possible relationships with other imaging markers as derived by positron emission tomography [89], structural MRI [90], and functional MRI [16, 91].

## 5 Conclusion

Based on MRI studies and a meta‐analytic approach, our results indicate higher iron levels in the putamen, caudate nucleus, and globus pallidus, but also the hippocampus and (less robust) in the thalamus, in AD patients. Together with a negative correlation of iron markers and MMSE scores in the globus pallidus, our work refines and provides further evidence for the notion that increased iron levels, especially in the basal ganglia, are a characteristic hallmark of AD, which can contribute to cognitive impairments. As such, our findings not only give novel insights into the pathogenesis of AD but also highlight the potential role of iron as a relevant marker in the diagnosis and possibly treatment of AD.

## Author Contributions

M.M. and N.B. conceived the study. M.M. and C.W. analyzed the data, and the results were interpreted together with N.B. M.M. and N.B. wrote the manuscript. All authors approved the manuscript.

## Conflicts of Interest

The authors declare no conflicts interests.

## Data Availability

Data are available at the Open Science Framework (OSF, https://tinyurl.com/3u9n6cwh).
